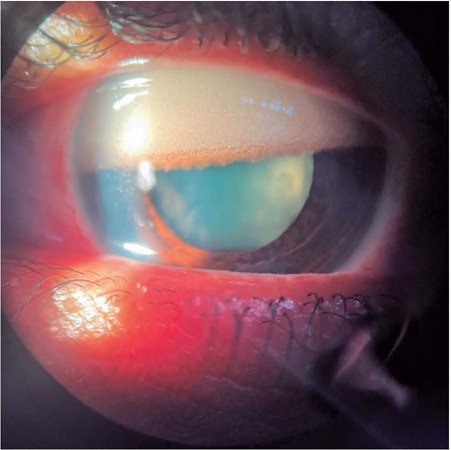# Emulsified silicone oil in the anterior chamber

**DOI:** 10.5935/0004-2749.2024-0058

**Published:** 2024-05-09

**Authors:** Laura G. Cyrino, Nicole B. M. Almeida, Newton Kara Junior

**Affiliations:** 1 Ophthalmology Department, Hospital das Clínicas, Universidade de São Paulo, São Paulo, SP, Brazil

Silicone oil (SO) may be used as a tamponade for complicated vitreoretinal diseases^(1)^. Due to its ability to displace aqueous humor from the retinal surface, SO maintains the adhesion between the retina and the retinal pigment epithelium^(2)^. Other properties of SO include intraocular inertness, transparency, and permanence until removal^(1)^. Currently, recommendations are to remove the SO as long-term placement in the vitreous cavity can result in complications. Emulsification of SO is a common cause of complications. Emulsified droplets have been found in both the anterior and posterior segments, optic chiasm, and brain, resulting in keratopathy, late-onset glaucoma, and optic neuropathy. Although the potential mechanisms vary, SO in the anterior chamber can result in contact between emulsified droplets with corneal endothelial cells, leading to inflammation in the trabecular meshwork and direct SO infiltration of angle structures^(1)^. Additionally, SO in the anterior chamber can lead to optical artifAacts such as bright reflex, reversed pattern of illumination, and interference fringes, due to contact with the endothelium^(3)^. However, the mechanism of SO emulsification remains unclear and is seemingly inevitable in some cases^(1)^.

**Figure:**